# Glioma biomarker analysis and drug targeting prediction based on ferroptosis-related genes

**DOI:** 10.1097/MD.0000000000048175

**Published:** 2026-04-10

**Authors:** Yang Zhang, Dan Wu, Yingjiang Gu, Hua Xiao

**Affiliations:** a Department of Neurosurgery, The Affiliated Traditional Chinese Medicine Hospital, Southwest Medical University, Luzhou, China.

**Keywords:** bioinformatics, biomarker, drug prediction, ferroptosis, glioma, immune infiltration

## Abstract

Glioma represents a significant challenge in neuro-oncology due to its highly invasive nature and poor prognosis. Recent studies indicate that ferroptosis, a form of regulated cell death, plays a crucial role in the pathogenesis of glioma. Modulating ferroptosis may offer a novel therapeutic approach for glioma treatment. This study aims to identify ferroptosis-related biomarkers and patterns of immune infiltration in glioma using bioinformatics techniques, and to predict potential therapeutic drugs targeting these biomarkers. Gene expression profiles (GSE16011, GSE50161) were retrieved from the GEO database, while ferroptosis-related genes were sourced from the FerrDb database. Intersection genes were identified using the CytoHubba algorithm and a consensus clustering method. Immune infiltration was assessed using the CIBERSORT algorithm. Functional enrichment analysis and gene set enrichment analysis were conducted with Gene Ontology and Kyoto Encyclopedia of Genes and Genomes databases. Drug targeting predictions were made based on identified hub genes. We identified 10 hub genes: TP53, RRM2, EZH2, CDKN1A, MYCN, KIF20A, BLM, GLS2, HMOX1, and GOT1. Gene Ontology and Kyoto Encyclopedia of Genes and Genomes analyses revealed that ferroptosis is primarily associated with apoptosis, reactive oxygen metabolism, the p53 signaling pathway, and immune regulation. Immune infiltration analysis showed significant differences in the expression of plasma cells, CD8 T cells, follicular helper T cells, activated NK cells, and macrophages between groups. qRT-PCR validation in glioma cell lines confirmed the differential expression of key ferroptosis-related genes, supporting the robustness of our bioinformatic findings. Furthermore, molecular docking demonstrates excellent binding properties between the drug components and key targets, and these key targets were further validated by PCR analysis in vitro. In summary, this study highlights the potential of ferroptosis-related biomarkers in glioma and suggests novel drug targets, providing a foundation for future therapeutic strategies.

## 1. Introduction

Glioma is a type of tumor originating from glial cells in the brain or spinal cord. It is one of the most common and aggressive forms of brain tumors. Gliomas are categorized based on their cell of origin into several subtypes, including glioblastoma (GBM), astrocytoma, oligodendroglioma, ependymoma, and oligoastrocytoma (mixed glioma). In recent years, the incidence of glioma has been steadily increasing. Statistics indicate that gliomas account for approximately 30% of all brain and central nervous system tumors and about 80% of all malignant brain tumors. The average annual incidence is 3 to 5 cases per 1,00,000 people. However, this rate increases with age, peaking in individuals aged 75 to 84. Men are more likely to develop gliomas than women, with an incidence rate approximately 1.5 times higher and a higher mortality rate.^[1]^

GBM is one of the most challenging malignant tumors, characterized by high mortality and recurrence rates. It is the most malignant type of glioma, accounting for approximately 59.2% of all gliomas.^[2]^ Standard treatment options for gliomas include surgery, radiotherapy, and chemotherapy. However, the average survival time for glioma patients after treatment is only 13 to 14 months, with a 5-year survival rate of merely 3% to 4%.^[3]^ Additionally, drug resistance and severe side effects pose significant challenges to effective glioma treatment. As a result, understanding the causes and developing targeted treatments for gliomas have become primary research focuses. Although the exact mechanisms of glioma pathogenesis remain unclear, several high-risk factors have been identified. High-dose ionizing radiation exposure is a major risk factor for glioma.^[4-6]^ Other factors such as age, gender, sleep patterns, alcohol consumption, the use of nonsteroidal anti-inflammatory drugs, and vitamin C intake have also been associated with glioma to varying extents.^[7-10]^ In recent years, research has increasingly focused on the genetic factors associated with gliomas. Investigations into glioma-related biomarkers have intensified, garnering significant attention from researchers. These biomarkers are valuable for monitoring the incidence of glioma and predicting patient prognosis. Tumor markers such as Ki67, RAB34, PPIC, EMP3, and CHI3L have been confirmed to be associated with gliomas.^[11,12]^ Recent studies suggest that glioma development is closely related to inflammation, cell pyroptosis, immune infiltration, and ferroptosis^[13-16]^。Therefore, discovering biomarkers linked to these mechanisms is crucial for advancing glioma treatment strategies.

Ferroptosis is an iron-dependent, novel type of programmed cell death distinct from apoptosis and autophagy.^[17]^ The hallmark feature of ferroptosis is the iron-dependent accumulation of oxidatively damaged phospholipids (lipid peroxides).^[18]^ The primary mechanism involves divalent iron or esteroxygenase catalyzing the peroxidation of highly expressed unsaturated fatty acids on the cell membrane, leading to cell death. Additionally, a reduction in GPX4, a core enzyme regulating the antioxidant glutathione system, is a characteristic of ferroptosis.^[19]^ Studies have shown that ferroptosis is closely related to a variety of diseases. In chronic liver disease models, ferroptosis is linked to stem cell damage, and inhibiting ferroptosis in stem cells can delay the progression of chronic liver disease. In rat models of Parkinson’s disease, ferroptosis is implicated in the disease’s development.^[20]^ Recent research indicates that ferroptosis is closely related to tumors and plays a significant regulatory role in tumor development.^[21]^ For instance, regulating ferroptosis can inhibit the progression of lung cancer and also has an important regulatory role in thyroid cancer.^[22,23]^ Although studies have demonstrated that ferroptosis is the most common type of programmed cell death in gliomas and is closely associated with glioma development, the specific molecular mechanisms remain unclear.^[24,25]^ Therefore, identifying ferroptosis-related targets in gliomas and elucidating their hierarchical molecular mechanisms will aid in understanding the etiology and treatment of gliomas and may provide new therapeutic targets.

Bioinformatics is an interdisciplinary field that combines biology, computer science, and statistics to develop and apply computational tools and technologies for processing and analyzing biological data. This approach enables the revelation of the structure, function, and dynamic changes of biological systems.^[26,27]^ Key areas of bioinformatics research include sequence analysis, genome assembly, gene expression analysis, protein structure prediction, and systems biology.^[28]^ In recent years, advancements in sequencing technology have positioned bioinformatics as a reliable method for researchers to deeply analyze various diseases. This study leverages bioinformatics techniques to uncover differences in gene expression between glioma and normal tissues, and to evaluate the signaling pathways and immune cell infiltration in gliomas based on ferroptosis-related genes. Additionally, we predicted potential drugs that may regulate ferroptosis and glioma through core targets. In summary, our research may provide new strategies for the diagnosis and treatment of glioma.

## 2. Materials and methods

### 2.1. Microarray collection and differential gene analysis

The bioinformatics data for glioma were sourced from the NCBI gene expression public database. We selected the GSE16011 and GSE50161 datasets for analysis. Probe IDs were converted into Gene symbols using the annotation information of the GPL8542 and GPL570 sequencing platforms, respectively. The 2 datasets were merged using the R software package (R Foundation for Statistical Computing, Vienna, Austria) inSilicoMerging,^[29]^ and Empirical Bayes was utilized to eliminate multi-batch effects.^[30]^ To perform differential gene analysis and identify significant differences in gene expression between glioma and normal brain tissue, we employed the “limma” (linear models for microarray data) package.^[31]^ Specifically, we acquired the expression profile dataset, utilized the lmFit function to conduct multiple linear regression, and further employed the eBays function to calculate the significance of differences for each gene. The threshold for identifying differential genes was set at a fold difference of 2.0 times and a *P*-value < .05. Positive numbers indicated upregulation of differentially expressed genes (DEGs), while negative numbers indicated downregulation. Visualization of the results of differential gene expression was achieved using volcano plots and heatmaps, providing a clear representation of the identified DEGs. Visualization of the results of differential gene expression was achieved using volcano plots and heatmaps, providing a clear representation of the identified DEGs.^[32]^

### 2.2. Acquisition of glioma genes and ferroptosis-related genes

To investigate disease targets and effects associated with glioma, we utilized “glioma” as the keyword to search 4 disease databases: GeneCards (https://www.genecards.org/), Comparative Toxicogenomics Database (CTD; https://ctdbase.org/), MalaCards (https://www.malacards.org/), and the DisGeNet database (https://www.disgenet.org/). These searches provided us with a comprehensive list of disease-related target proteins.^[33]^ Genes related to ferroptosis were obtained from the FerrDb database. By taking the intersection of the gene sets retrieved from GeneCards, CTD, MalaCards, DisGeNet, the DEGs, and ferroptosis-related genes, we identified a set of genes that were potentially important for the occurrence and development of glioma. These genes were preliminarily identified as key target genes.

### 2.3. Forest model and nomogram model construction

In this study, Lasso analysis was employed to preliminarily screen the intersection genes and predict the likelihood of glioma occurrence. LASSO, which stands for Least Absolute Shrinkage and Selection Operator, is a linear regression method that utilizes L1 regularization. The application of L1 regularization results in some of the learned feature weights being set to 0, thereby achieving sparsity and feature selection. The candidate differentially expressed ferroptosis genes were integrated into the model and analyzed using the LASSO algorithm to identify characteristic genes associated with glioma. These identified genes were subsequently utilized for predicting the incidence of glioma, and receiver operating characteristic analysis was performed to assess the predictive performance.

### 2.4. Consistency cluster analysis

The expression profiles of glioma ferroptosis-related genes were subjected to consensus clustering analysis using the ConsensusClusterPlus package. This clustering method, commonly employed in tumor molecular classification studies, aids in determining the optimal number of clusters. The procedure involves resampling the original dataset to generate multiple sub-datasets, each of which undergoes separate clustering. Subsequently, the clustering results from all sub-datasets are combined to calculate the consistency index (consensus index, ranging from 0 to 1) of sample clustering. A higher index indicates more stable clustering. The selection of the optimal number of clusters is guided by 2 principles:

1. In the cumulative distribution function curve of the consistency index, the cluster number with the flattest middle section of the curve is chosen. A flatter curve corresponds to a lower proportion of fuzzy clusters, indicating fewer sample pairs with unclear assignments in different sub-dataset clustering runs.
2. Within the consistency matrix, high consistency indices are sought for samples within a subgroup, while low consistency indices are desired between subgroups.

The black stripes in the Tracking Plot represent the samples, illustrating their classification under different *k* values. Different color blocks denote distinct classifications. Instability in sample classification under different *k* values indicates unstable clustering. This study examined sample clustering consistency across a range of cluster numbers (*k* = 2–7) using the PAM (Partitioning around medoid) clustering method, with sample distances calculated using the Pearson method.^[34]^。

### 2.5. PPI network establishment

Protein–protein interactions (PPIs) are fundamental to cellular biological processes and are crucial for understanding cellular physiology in both normal and diseased states. In this study, we utilized the STRING database (http://string-db.org/) to perform PPI network analysis on the identified intersection gene set (glioma mechanism gene set). The species was restricted to “Homo sapiens,” and the confidence value threshold was set to >0.4. The PPI network was constructed using Cytoscape software (version 3.9.1).^[35]^ Additionally, the CytoHubba algorithm, a plugin for Cytoscape, was employed to build the glioma ferroptosis mechanism gene regulatory network.^[36]^ Through further screening, 10 key hub genes were identified. Gene co-expression analysis of these hub genes was conducted using the STRING database, with results displayed based on RNA expression patterns and protein co-regulation scores provided by ProteomeHD.

### 2.6. Enrichment analysis

To analyze the ferroptosis-related signaling pathways and mechanisms of action in glioma, the intersection genes were subjected to Gene Ontology and Kyoto Encyclopedia of Genes and Genomes enrichment analysis.^[37]^ The results of these analyses are presented in various graphical formats to illustrate the significant biological processes, molecular functions, cellular components, and pathways involved.

### 2.7. Identification and association of disease immune infiltrating cells

The immune microenvironment comprises immune cells, inflammatory cells, fibroblasts, mesenchymal cells, various cytokines, and chemokines.^[38]^ Analyzing immune cell infiltration is crucial for predicting disease progression and treatment responses. The CIBERSORT algorithm, which deconvolutes gene expression profiles via linear support vector regression, is used to estimate the number of immune cells in samples based on RNA sequencing data.^[39]^ This algorithm calculates the types of immune cells in patients with different immune patterns in the dataset and uses a stacked graph to display the immune cell composition of these patients. Finally, statistical differences between various immune cells are depicted using a box plot to highlight the differential abundance.

### 2.8. Drug prediction based on hub genes

Using the 10 pyroptosis hub genes identified in bladder cancer, we leveraged the key gene set prediction drug analysis tool available on the BioCloud platform to forecast potential therapeutic drug components for bladder cancer. We selected the top 6 key active ingredients and cross-referenced them in the DrugBank and Traditional Chinese Medicine Systems Pharmacology databases. The mechanisms of action of these related drugs were then analyzed to understand their potential therapeutic effects.

### 2.9. qRT-PCR

Human glioma cell lines (U251) and a normal human astrocyte cell (HA1800) were obtained from the Procell (Wuhan, China). The experiment was divided into control (normal human astrocyte cell) group and glima (Human glioma cell lines) group. Cells were cultured in DMEM supplemented with 10% fetal bovine serum (Gibco, USA) and 1% penicillin-streptomycin under standard conditions (37°C, 5% CO_2_).

Total RNA was extracted using TRIzol reagent (Invitrogen) according to the manufacturer’s instructions. cDNA synthesis was performed using the PrimeScript™ RT reagent kit (Takara, Japan). qRT-PCR was carried out with SYBR® Premix Ex Taq™ II (Takara) on a CFX96 Real-Time PCR Detection System (Bio-Rad). Relative gene expression levels were calculated using the 2^−ΔΔCt^ method. GAPDH served as an internal control. The primers used in the experiment are as follows:

EZH2-Forward: AGTGTGGAGGAGAACGCTGA,

EZH2-Reverse: GTGCAGCAAGAGCAGAGAGT;

TP53-Forward: CCCCTCCTGGCCCCTGTCATCTT,

TP53-Reverse: GCAGCGCCTCACAACCTCCGTCAT;

RRM2-Forward: GGAGAGGAAGATGTGGAGGAAG,

RRM2-Reverse: AAGGCTGCATTTGATGAAGAGG;

CDKN1A-Forward: CCTGTCACTGTCTTGTACCCT,

CDKN1A-Reverse: GCGTTTGGAGTGGTAGAAATCTG;

MYCN-Forward: GCTGCGCACGCTGCAAGGAATA,

MYCN-Reverse: CGCTCCGGACCGCTTGTT;

KIF20A-Forward: ATCCTGAAGACCATGGTCCTG,

KIF20A-Reverse: GGCTCCTCTTGTCTTCTGTCC;

BLM-Forward: GAGGCTGGAGAAGTGAGGAG,

BLM-Reverse: TTTCCTGAGCCAGGTAGGAG;

GLS2-Forward: GAGGTGACGATGAACGCTTTG,

GLS2-Reverse: CTCTGGCTCCTCATAGGCTTC;

HMOX1-Forward: GTGCACATCCGTGCAGAGA,

HMOX1-Reverse: GGTGTAAGGACCCATCGGAG;

GOT1-Forward: TGTGGCAGAGAGCTTTGACTC,

GOT1-Reverse: AGCTGGATGTCGATGGAGTGT;

GAPDH-Forward: GACAGTCAGCCGCATCTTCT,

GAPDH-Reverse: TTAAAAGCAGCCCTGGTGAC.

### 2.10. Statistical analysis

All data were analyzed using GraphPad Prism 9.0 and presented as mean ± standard deviation. Differences in gene expression levels between control group and glioma group were evaluated using unpaired 2-tailed Student *t* test. A *P*-value < .05 was considered statistically significant. All experiments were performed with at least 6 biological replicates to ensure robustness and reproducibility.

## 3. Result

### 3.1. Microarray differential gene analysis results

**Figure 1.**
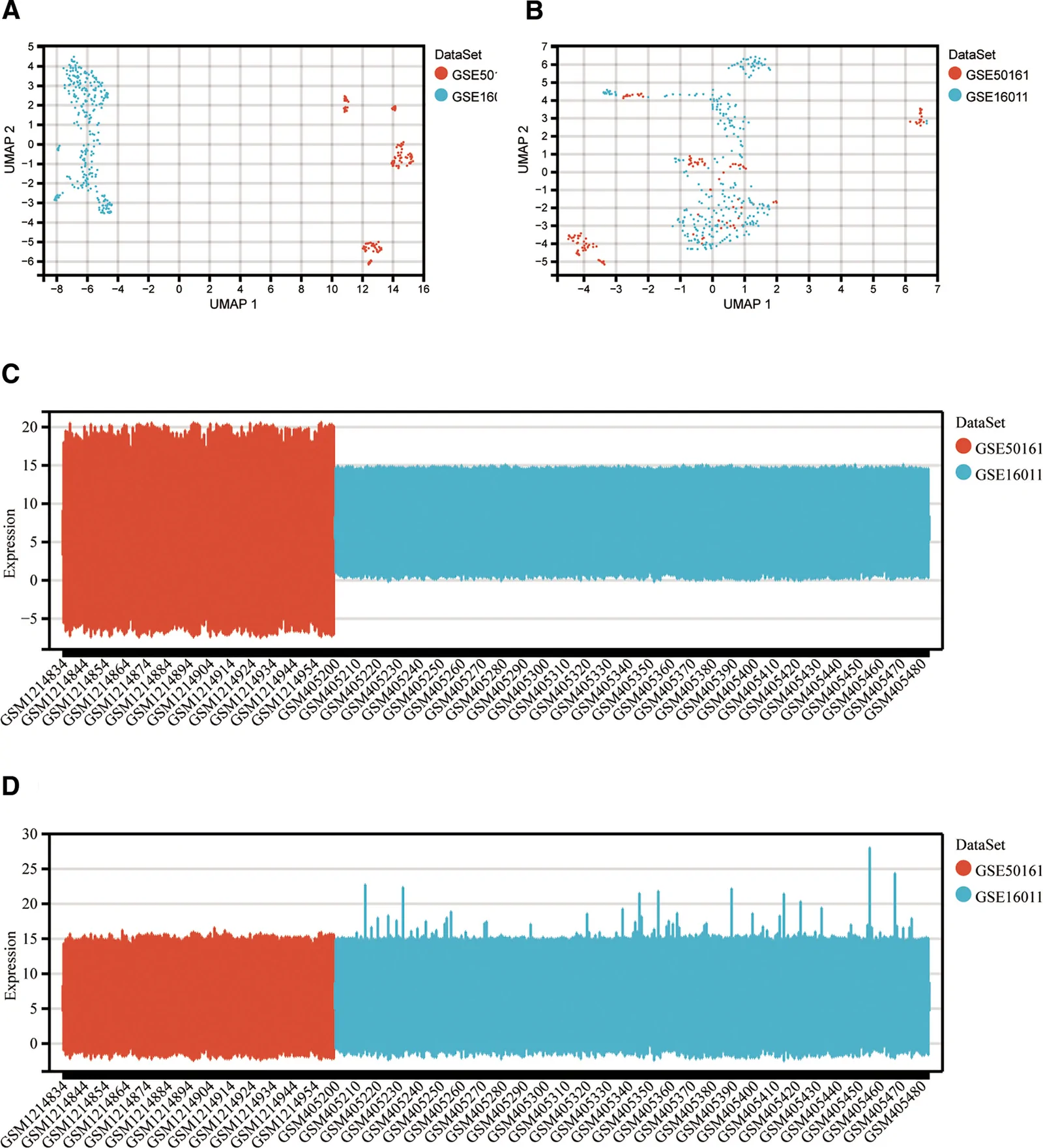
Batch effect removal diagram: (A) UMAP diagram showing that before batch effect removal, samples from each dataset cluster separately, indicating the presence of a batch effect. (B) UMAP diagram after batch effect removal, with samples from different datasets clustering together, indicating successful removal of the batch effect. (C) Distribution of sample data before batch effect removal, showing significant differences among datasets, confirming the batch effect. (D) Distribution of sample data after batch effect removal, showing consistent data distribution across datasets, with similar means and variances. UMAP = Uniform Manifold Approximation and Projection.

**Figure 2.**
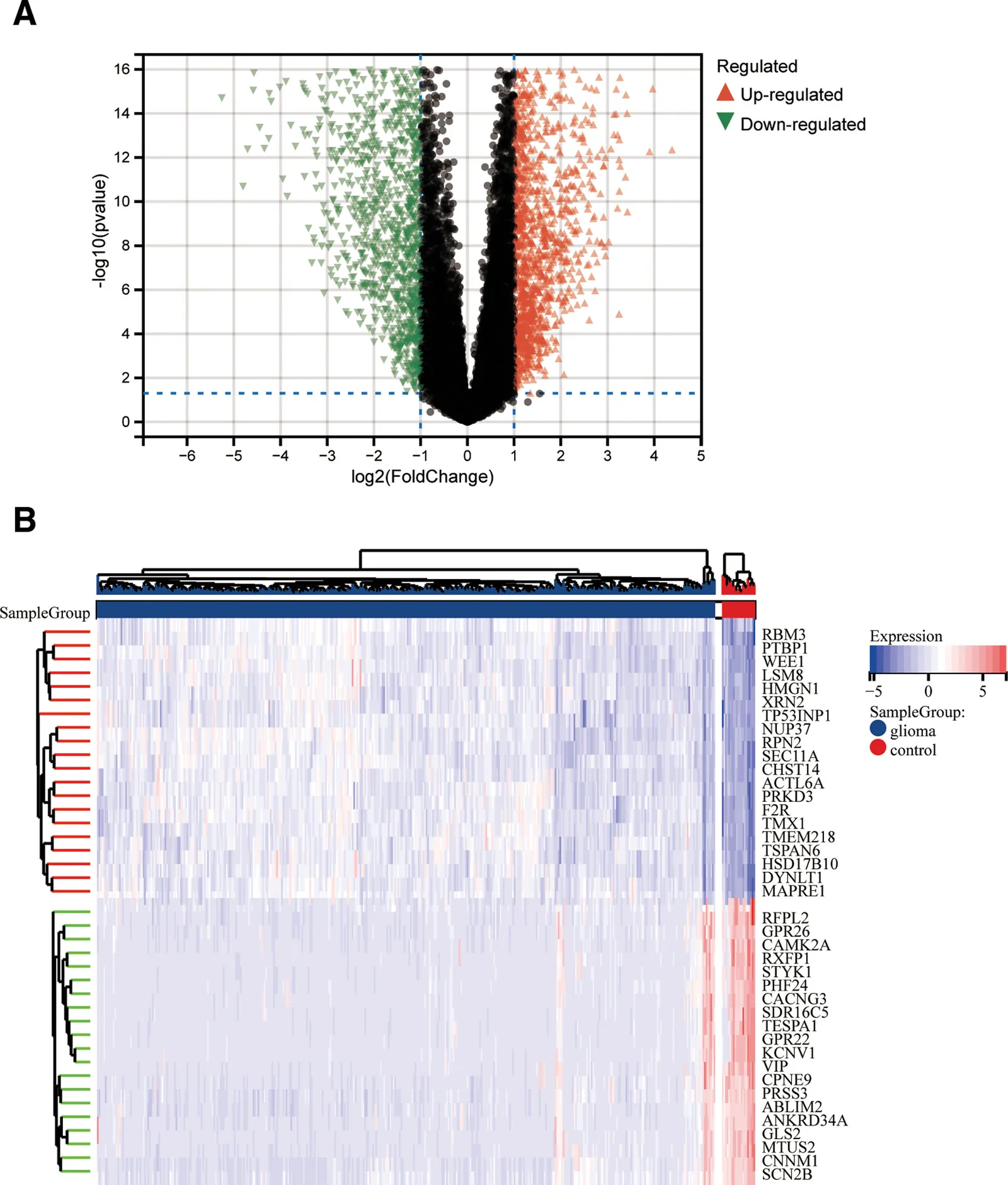
Differential gene analysis: (A) volcano plot of differentially expressed genes based on the original data matrix. (B) Heat map of differentially expressed genes based on the original data matrix.

The GSE16011 dataset analyzed in this study includes 278 glioma samples and 8 normal brain tissue samples, while the GSE50161 dataset includes 117 glioma samples and 13 normal brain tissue samples. After ID conversion and merging, the expression matrix of Gene Symbol was obtained (Table S1, Supplemental Digital Content, https://links.lww.com/MD/R649). Figure 1 illustrates the results before after merging the datasets. Differential analysis between glioma samples and normal control samples revealed 4092 DEGs out of 16,811 genes, with 1484 upregulated and 1677 downregulated genes (Table S2, Supplemental Digital Content, https://links.lww.com/MD/R649). Figure 2 presents the volcano plot and heat map of these DEGs.

### 3.2. Interaction results of glioma genes and ferroptosis-related genes

**Figure 3.**
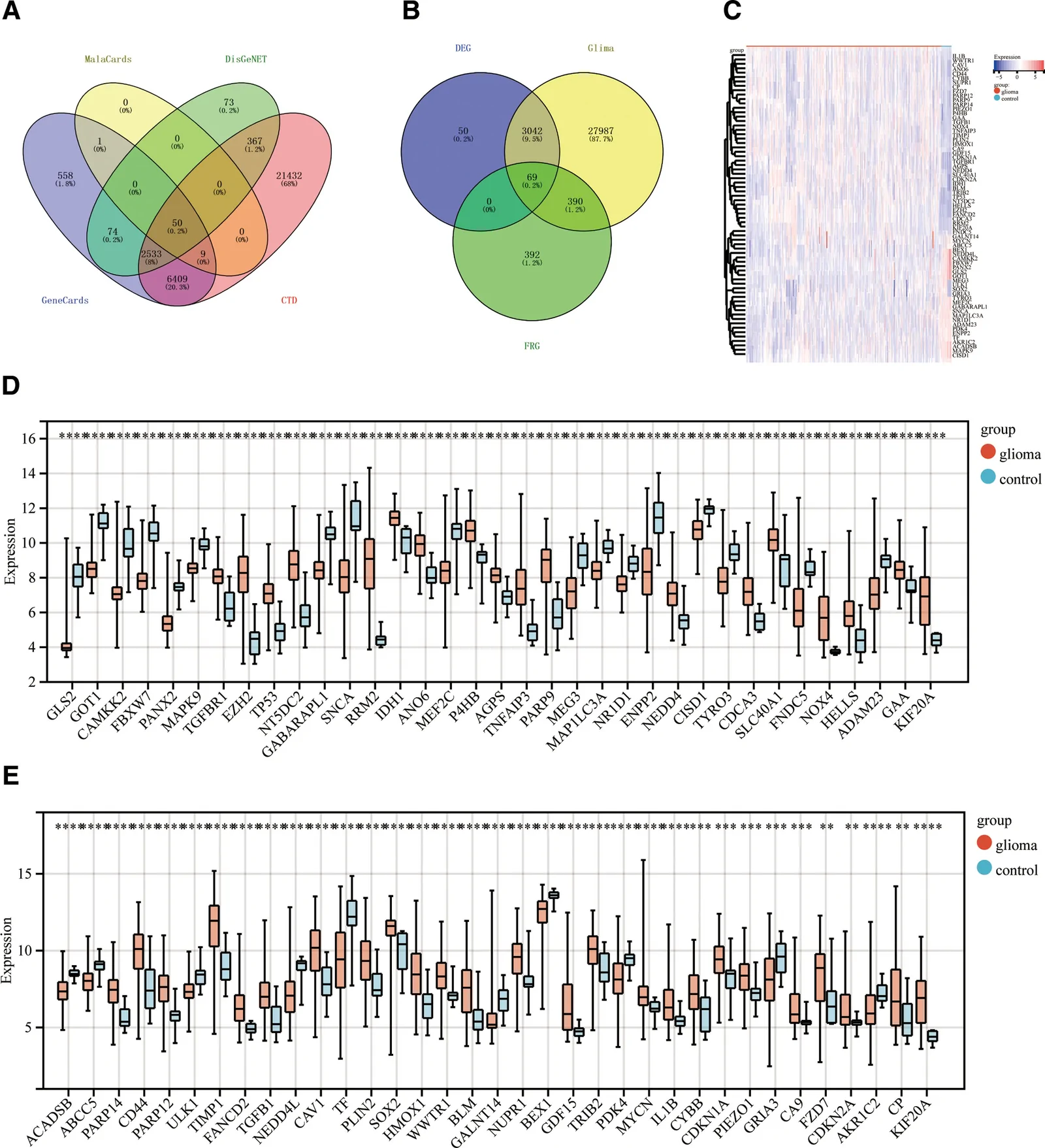
Analysis of glioma disease-related targets and intersection of ferroptosis-related genes: (A) bar chart showing the number of glioma-related targets in different datasets. (B) Venn diagram showing the intersection of differentially expressed genes, disease-related genes, and ferroptosis-related genes. (C) Heat map of intersecting genes. (D, E) Box plots of intersecting genes.

We identified 9634 glioma-related genes from the GeneCards disease database, 60 from the MalaCards database, 3097 from the DisGeNET database, and 30,800 from the CTD database. After merging these gene sets and removing duplicates, we obtained a total of 31,488 glioma-related genes (Fig. 3A, Table S3, Supplemental Digital Content, https://links.lww.com/MD/R649). From the FerrDb database, we obtained 851 ferroptosis-related genes (Table S4, Supplemental Digital Content, https://links.lww.com/MD/R649). The intersection of glioma-related genes, DEGs, and ferroptosis-related genes resulted in the identification of 69 glioma ferroptosis-related genes (Fig. 3B, Table S5, Supplemental Digital Content, https://links.lww.com/MD/R649). Figure 3C presents an expression heat map of these intersection genes, while Figure 3D, E display box plots of their relative expression levels.

### 3.3. Risk model construction

**Figure 4.**
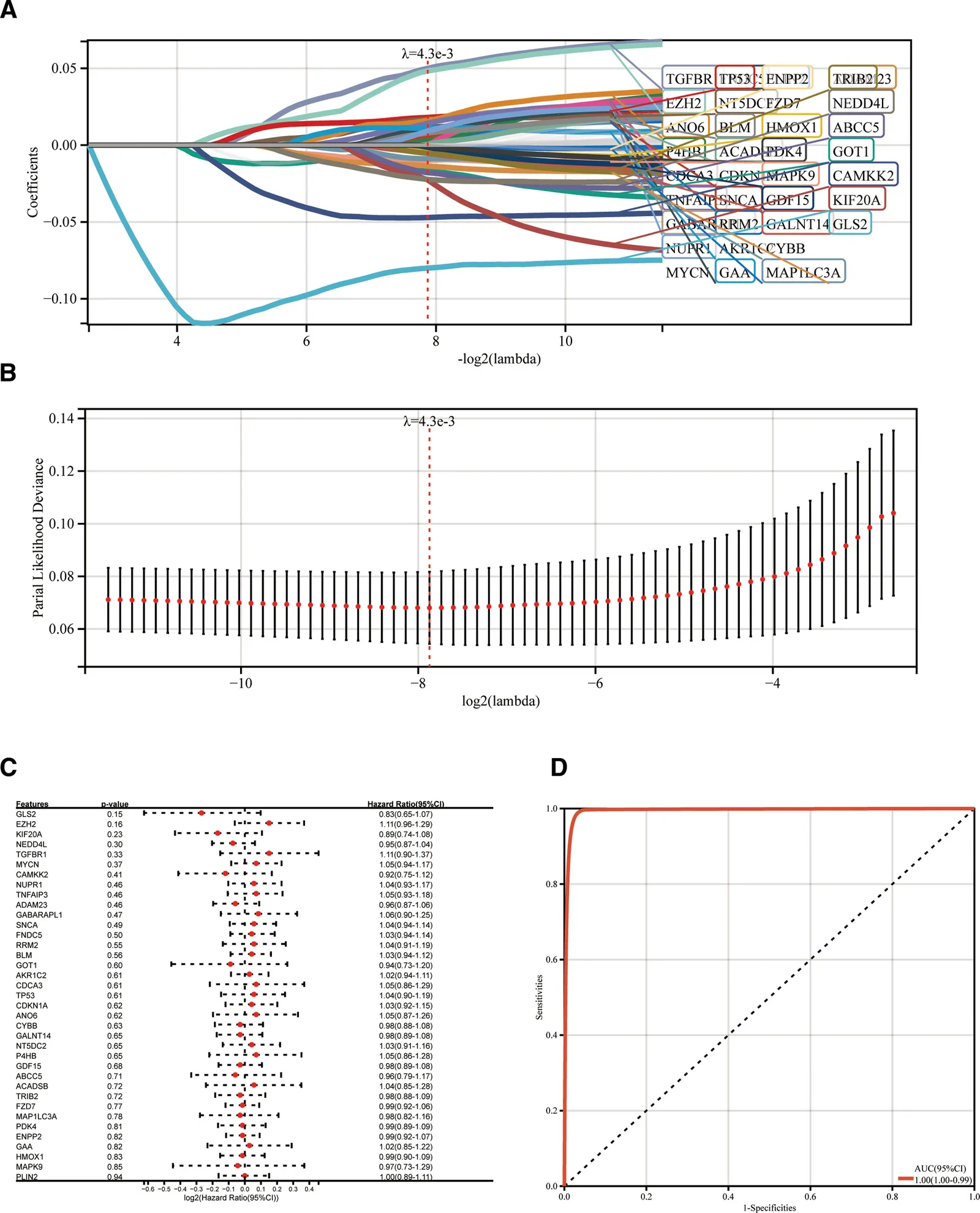
Construction of a risk model: (A, B) selection of 37 characteristic genes using the LASSO algorithm. (C) Forest plot displaying the expression of 37 characteristic genes. (D) ROC curve for the risk model. ROC = receiver operating characteristic.

To identify key genes significantly impacting glioma, we utilized the LASSO (Least Absolute Shrinkage and Selection Operator) algorithm on the intersection genes. Using the R package glmnet, we integrated survival status and gene expression data to perform regression analysis. A 10-fold cross-validation was set up to ensure the optimal model, with the Lambda value set to 0.0042, resulting in the identification of 37 key genes (Fig. 4A, B). Based on the coefficients of these 37 feature genes (Fig. 4C), we employed the R package survival to integrate data on survival time, survival status, and the expression of the 37 feature genes. The Cox proportional hazards model was used to evaluate the prognostic significance of these genes in 414 samples. Specifically, we calculated the patients’ risk scores and correlated them with the expression and status of the 37 key genes. The roc function of the pROC package was used to perform receiver operating characteristic analysis, and the ci function of pROC was used to evaluate the AUC (Area Under the Curve) and confidence intervals, resulting in the final AUC results (Fig. 4D).

### 3.4. Consistency cluster analysis results

**Figure 5.**
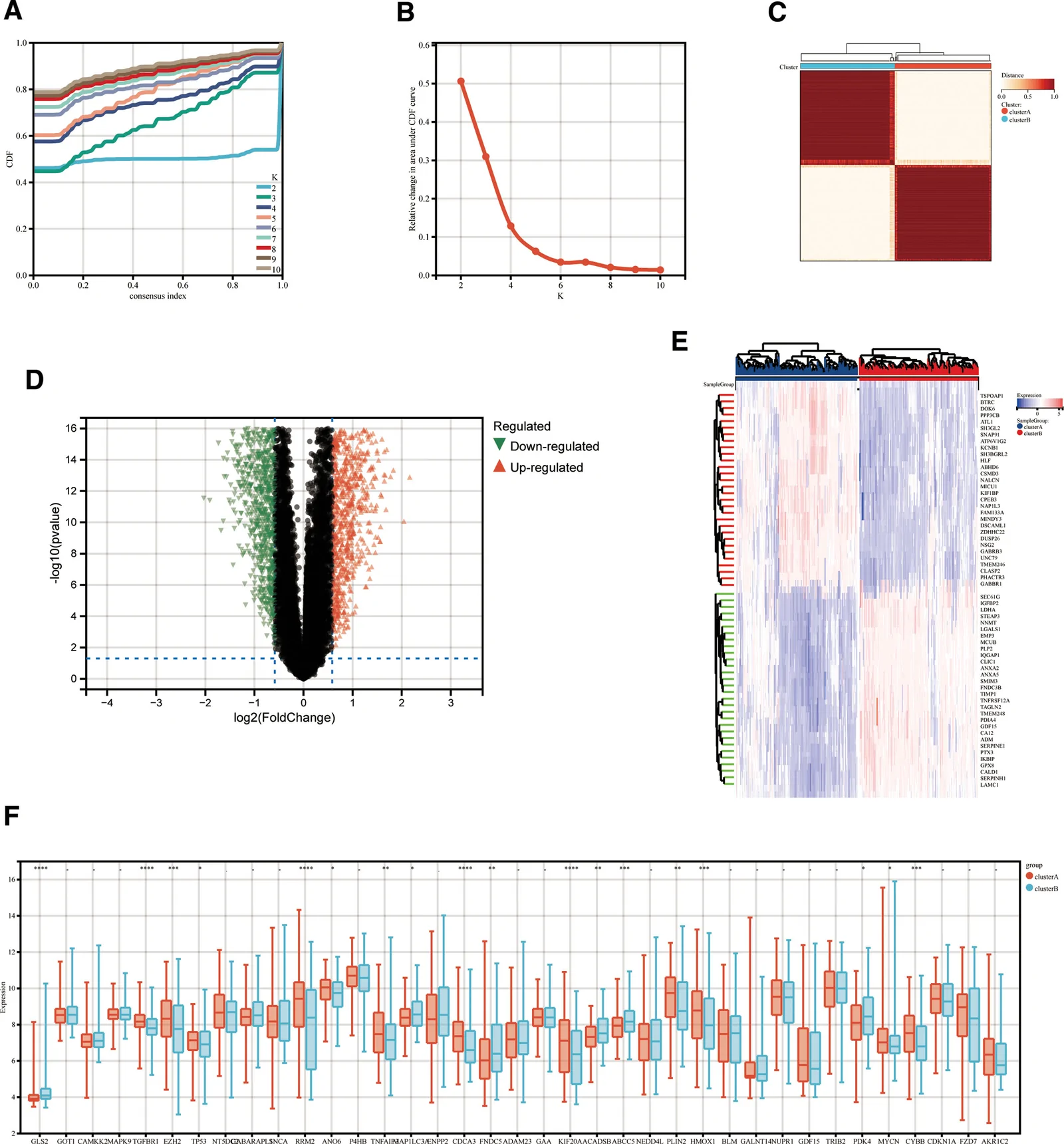
Consistency clustering analysis: (A) cumulative distribution function (CDF) curve. (B) Changes in correlation of the CDF curve. (C) Clustering diagram of ferroptosis-related genes. (D) Volcano plot of ferroptosis-related genes after clustering. (E) Heat map of ferroptosis-related genes after clustering. (F) Box plot of ferroptosis-related genes after clustering. CDF = cumulative distribution function.

Utilizing the expression data of 69 ferroptosis-related genes in glioma obtained from previous analyses, we conducted a cluster analysis on the patient samples. The results, displayed in Figure 5A, B, indicate that the cumulative distribution function curve flattens most distinctly when the number of clusters, *k* = 2. This suggests that the number of sample pairs with ambiguous distribution is minimized during the clustering process. The consistency matrix further confirms this, showing a higher clustering consistency index for samples within the same subtype and a lower index between different subtypes when *k* = 2 (Fig. 5C). Thus, we determined *k* = 2 as the optimal number of clusters, dividing glioma patients into 2 ferroptosis subgroups: cluster A and cluster B, with 209 and 205 samples respectively (detailed in Table S6, Supplemental Digital Content, https://links.lww.com/MD/R649). To elucidate the differences in ferroptosis characteristics between these subtypes, we performed a differential analysis on the expression levels and comprehensive scores of their ferroptosis genes. The results, shown in Figure 5D, reveal significant differences in the ferroptosis gene expression levels between the 2 subpopulations (Wilcoxon test, *P* < .001; Fig. 5E).

### 3.5. PPI network results

**Figure 6.**
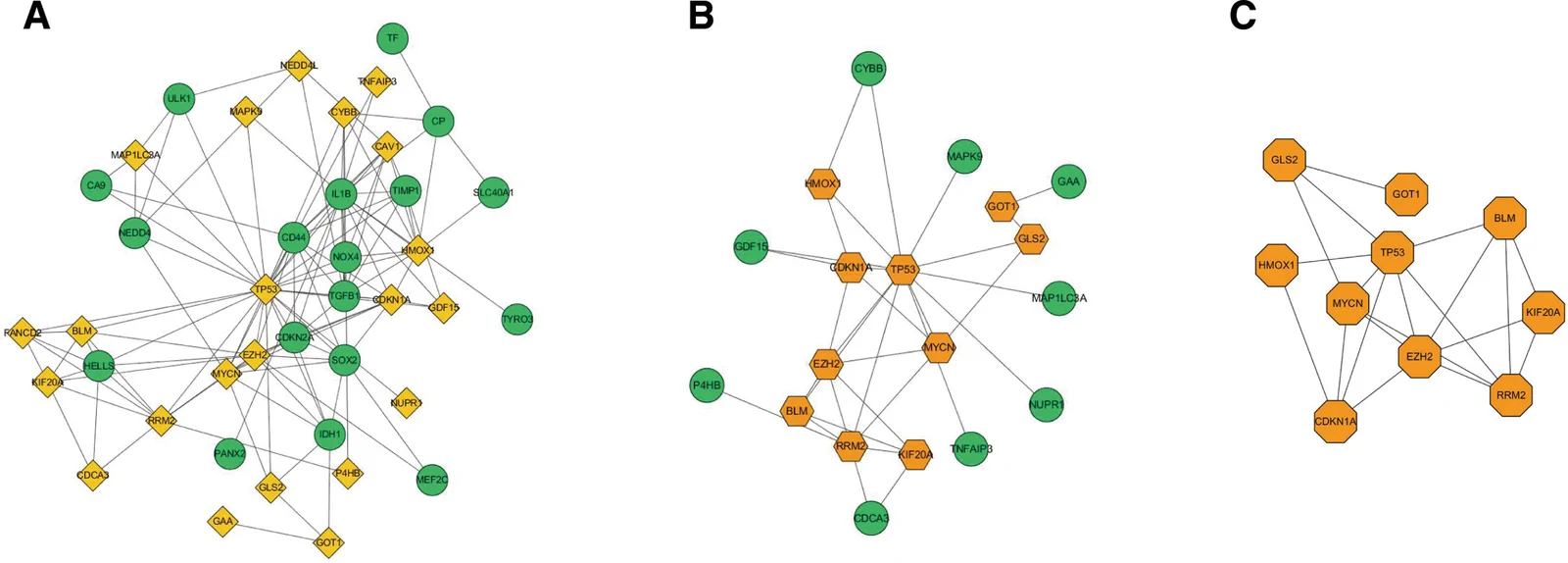
PPI network diagram: (A) interaction network of 69 intersecting genes. (B) Interaction network of 37 characteristic genes. (C) Interaction network of 10 hub genes.

To delve into the interplay among differentially expressed ferroptosis genes, we constructed PPI networks focusing on both the intersection genes and characteristic genes. Visualized in Cytoscape, the PPI network derived from intersection genes exhibited 370 pairwise interactions, with TP53 closely associated with 32 intersection genes, and CDKN1A linked to 31 intersection genes (Fig. 6A). The key gene PPI network showcased 92 pairwise interactions; TP53 was closely associated with 17 intersection genes, while CDKN1A was linked to 7 intersection genes (Fig. 6B). Following additional screening with the CytoHubba algorithm, we identified 10 hub genes related to glioma ferroptosis (Fig. 6C). These hub genes comprise TP53, RRM2, EZH2, CDKN1A, MYCN, KIF20A, BLM, GLS2, HMOX1, and GOT1. Co-expression scores, derived from RNA expression patterns and protein co-regulation via ProteomeHD, underscored the significance of these 10 hub genes, indicating substantial co-expression with other genes and a mutually reinforcing effect. Among these hub genes, TP53 exhibited the most frequent co-expression with other genes, suggesting its potential as the core target of ferroptosis in glioma.

### 3.6. Enrichment analysis results

**Figure 7.**
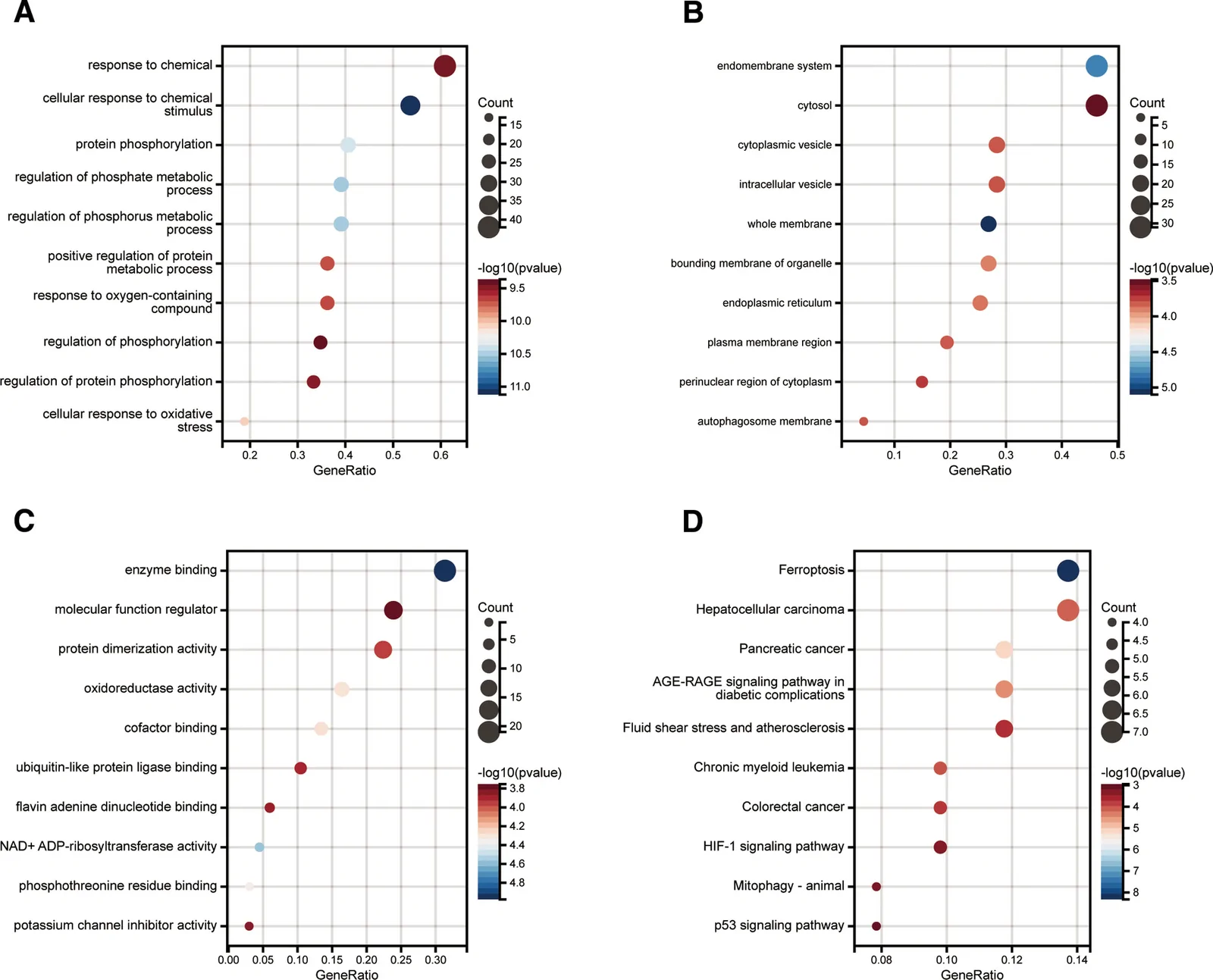
GO and KEGG enrichment analysis: (A–C) results of GO analysis showing biological processes (BP), cellular components (CC), and molecular functions (MF) respectively. (D) Results of KEGG pathway analysis. BP = biological process, CC = cellular component, GO= Gene Ontology, KEGG= Kyoto encyclopedia of genes and genomes, MF = molecular function.

The enrichment analysis of the 10 core glioma ferroptosis-related genes yielded comprehensive insights. The Gene Ontology enrichment outcomes encompassed a total of 358 entries, distributed across 245 biological process, 39 cellular component, and 74 molecular function categories. Notable biological process categories included responses to x-ray and ionizing radiation, DNA damage response, signal transduction by p53 class mediator resulting in transcription of p21 class mediator, replicative senescence, and regulation of reactive oxygen species metabolic processes. Cellular component enrichment highlighted key cellular components such as the nucleus, nucleolus, nucleoplasm, and chromatin silencing complex, while molecular function analyses underscored functions like activator activity, p53 binding, and ATP-dependent DNA/DNA annealing activity. In addition, Kyoto Encyclopedia of Genes and Genomes pathway analysis revealed enrichment in 82 pathways, with significant involvement in Ferroptosis, the p53 signaling pathway, and various cancer types including thyroid, bladder, and endometrial cancer. Other enriched pathways included those related to basal cell carcinoma, microRNAs in cancer, central carbon metabolism in cancer, and platinum drug resistance (Fig. 7D). These findings collectively suggest a close association between glioma pathogenesis and ferroptosis, apoptosis, reactive oxygen metabolism, the p53 signaling pathway, and immune regulation, shedding light on potential therapeutic targets and mechanisms underlying glioma development.

### 3.7. Analysis of immune infiltration results

**Figure 8.**
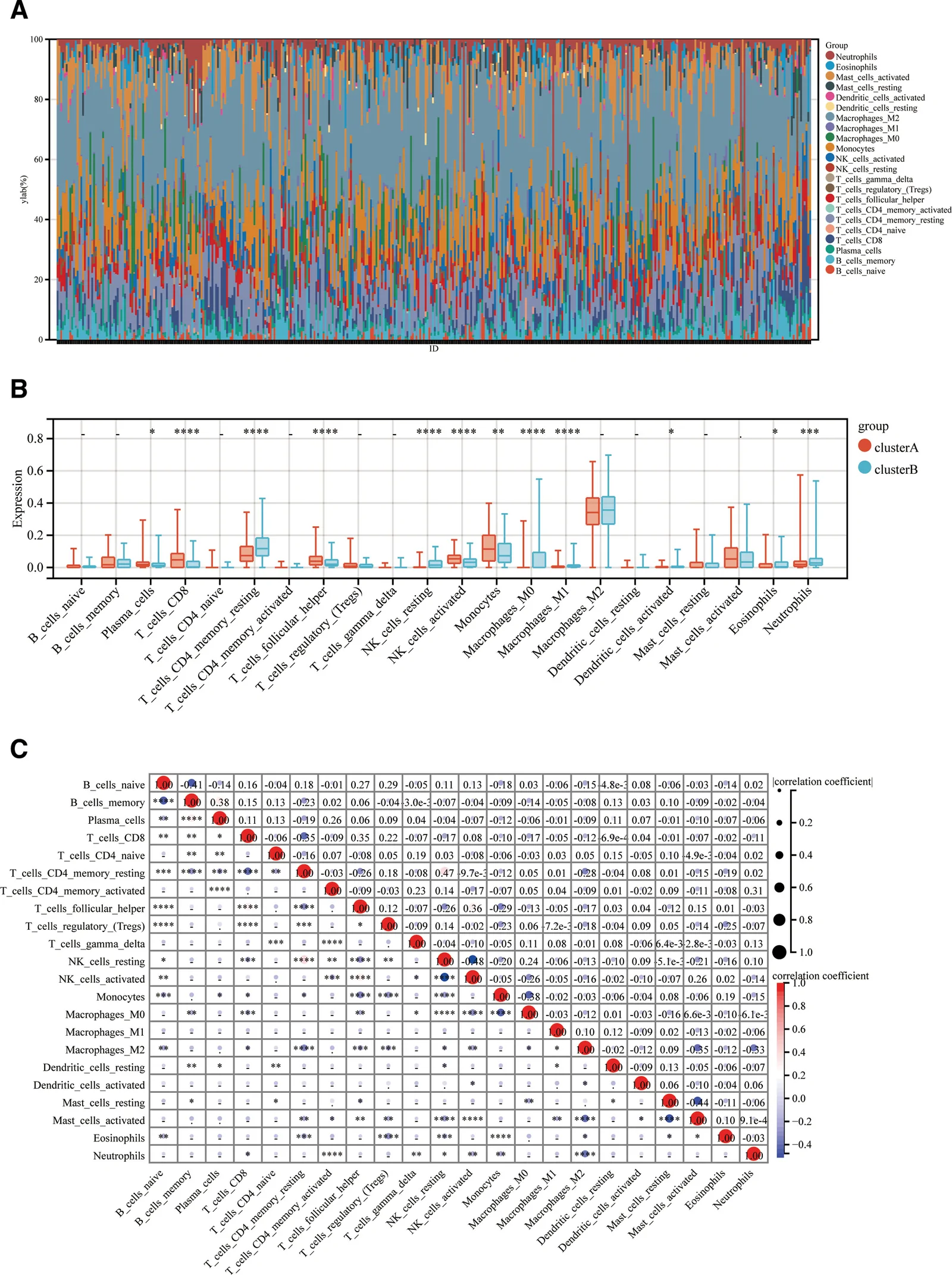
Immune infiltration analysis: (A) expression levels of immune cells in each sample. (B) Box plot comparing immune cell expression between groups. (C) Correlation analysis of immune cells.

Utilizing the CIBERSORT algorithm, we assessed immune cell infiltration levels across 2 distinct immune profiles. The analysis revealed significant differences in the abundance of various immune cell populations between group A and group B patients. Group A patients exhibited markedly higher levels of plasma cells, CD8 T cells, follicular helper T cells, activated NK cells, and macrophages compared to group B patients (Fig. 8A). Conversely, the levels of resting memory CD4 T cells, resting NK cells, M0 macrophages, M1 macrophages, dendritic cells, eosinophils, and neutrophils were significantly lower in group A compared to group B (Fig. 8B). Further examination of the correlation between immune cell populations unveiled several notable associations. Activated NK cells showed a negative correlation with resting NK cells, while memory B cells exhibited a negative correlation with activated B cells. Moreover, resting memory CD4 T cells displayed a positive correlation with resting BK cells (Fig. 8C). These findings underscore the intricate interplay between different immune cell populations and their potential implications for glioma pathogenesis and treatment response.

### 3.8. Targeted drug prediction

**Figure 9.**
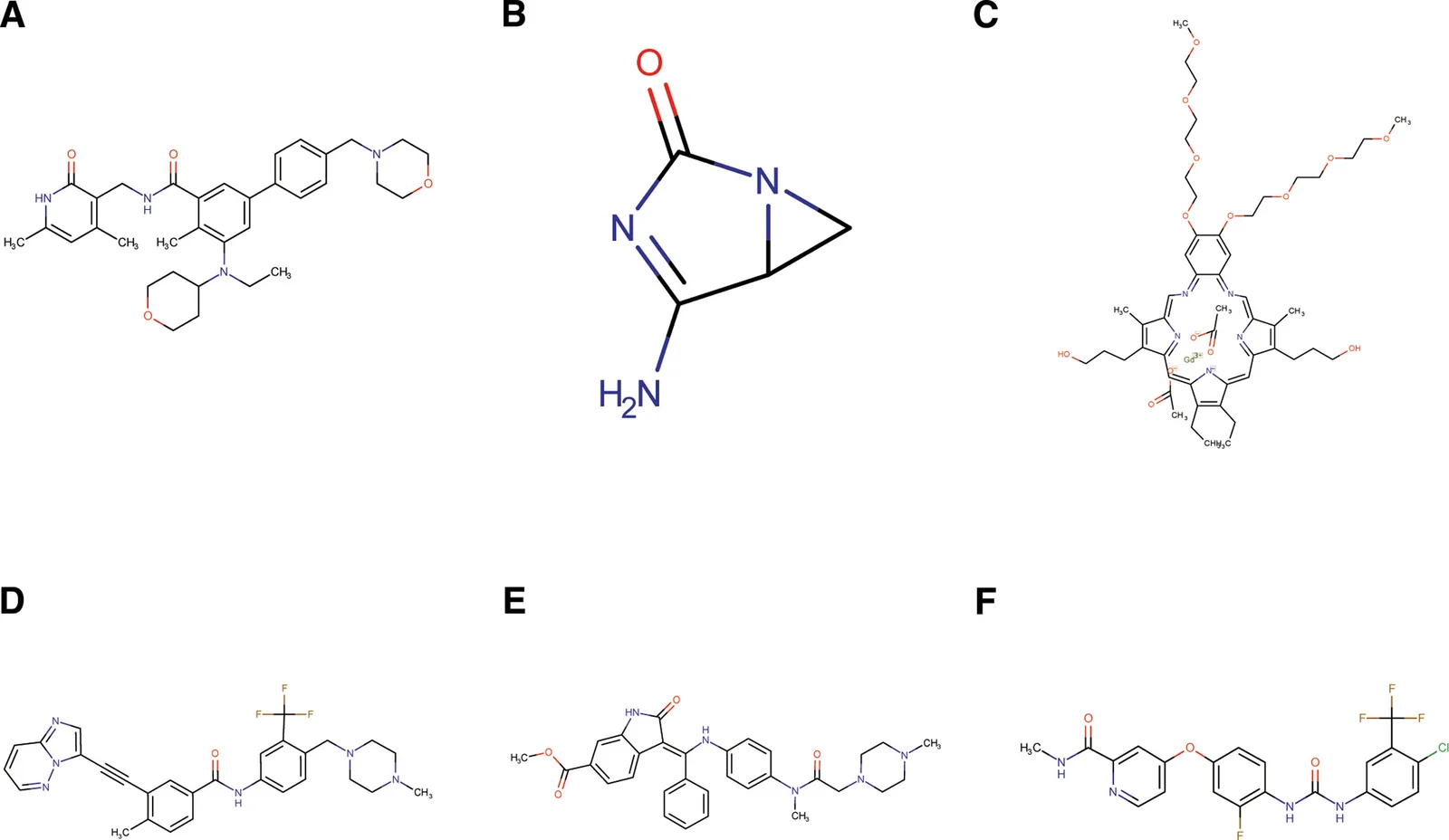
Drug targeting prediction: (A–F) molecular structures of Tazemetostat, Imexon, Motexafin gadolinium, Ponatinib, Nintedanib, and Regorafenib, respectively.

In an effort to unveil potential therapeutic avenues leveraging ferroptosis-related molecules in glioma treatment, we initiated drug prediction analysis based on 10 ferroptosis hub genes. Employing the key gene set prediction drug analysis tool, we predicted 43 relevant compounds. Subsequently, through a meticulous process involving distance sorting combined with literature scrutiny, we identified 6 prominent compounds with promising therapeutic potential. These compounds are Tazemetostat, Imexon, Motexafin gadolinium, Ponatinib, Nintedanib, and Regorafenib (Fig. 9). This focused exploration of targeted drugs highlights the feasibility of leveraging ferroptosis-related mechanisms in glioma therapy and underscores the importance of further investigation into these compounds for potential clinical application.

### 3.9. qRT-PCR validation of hub genes

**Figure 10.**
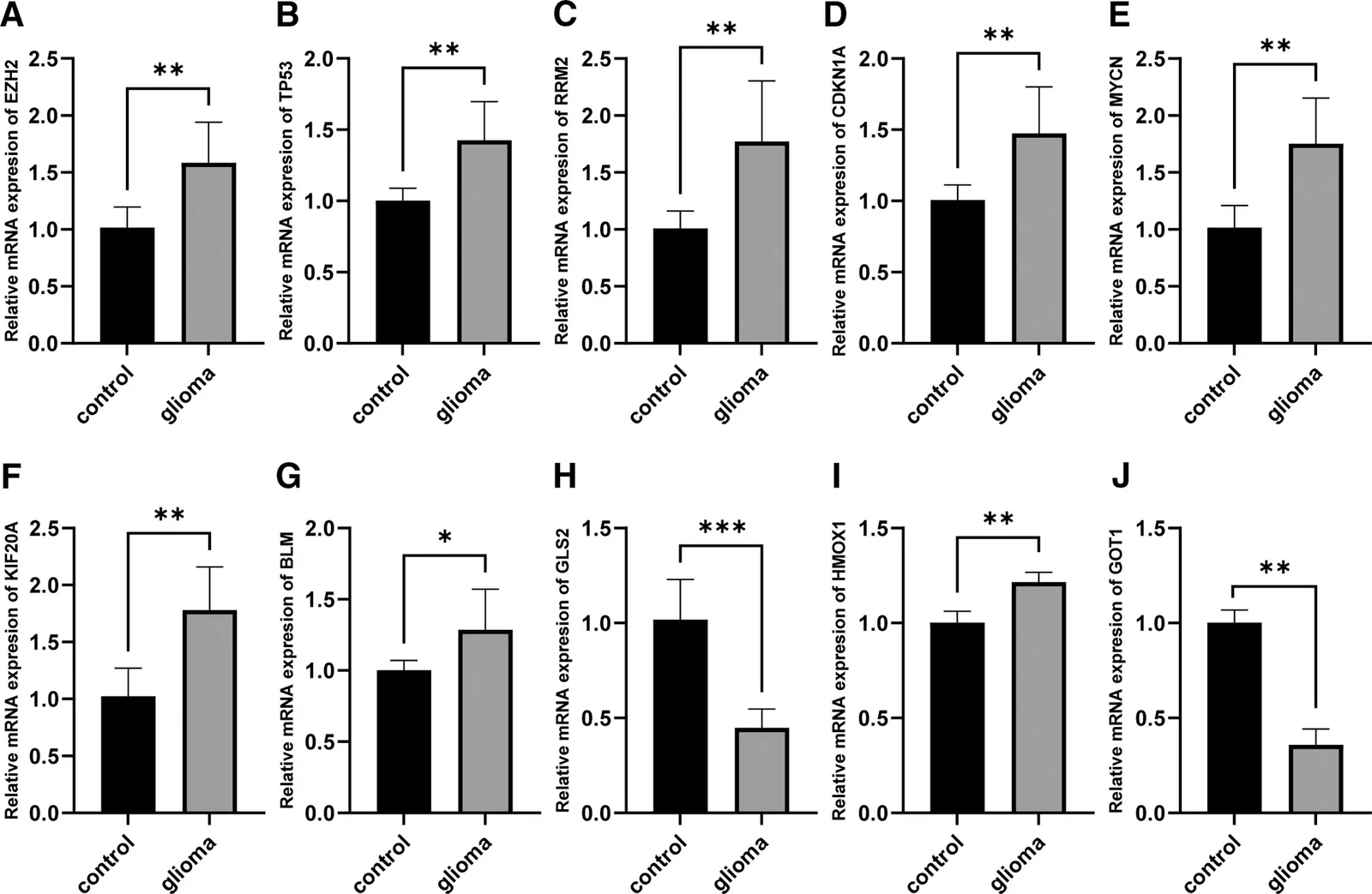
qRT-PCR validation of hub genes: (A–J) relative expression of hub gene EZH2, TP53, RRM2, CDKN1A, MYCN, KIF20A, BLM, GLS2, HMOX1, and GOT1 mRNA in the control group and glioma group.

Among the 10 ferroptosis-related hub genes, EZH2, TP53, RRM2, CDKN1A, MYCN, KIF20A, BLM, and HMOX1 exhibited significantly higher mRNA expression levels in glioma cells compared to normal astrocytes (*P* < .05). In contrast, GLS2 and GOT1 were markedly downregulated in glioma cells (*P* < .05), consistent with their potential tumor-suppressive roles in ferroptosis regulation (Fig.10). These findings support the robustness of our computational predictions and provide experimental evidence for the relevance of ferroptosis-related genes in glioma pathogenesis.

## 4. Discussion

Glioma represents a formidable challenge in the realm of neuro-oncology, characterized by its formidable invasiveness and dire prognosis. Among gliomas, GBM stands out as the most prevalent and devastating subtype.^[40]^ Epidemiological evidence underscores its predilection for older individuals and males.^[1]^ Despite advances in therapeutic modalities such as surgery, radiation, and chemotherapy, outcomes remain bleak, with a median survival of approximately 15 months and a dismal 5-year survival rate below 10%.^[3]^ Recent advancements in our understanding of tumor biology have shed light on ferroptosis, an emerging cell death mechanism that holds significant promise in oncology. This study endeavors to delve into the potential implications of ferroptosis-related genes in glioma pathogenesis and therapeutics. By conducting a comprehensive analysis, we aim to unveil novel biomarkers and targeted therapeutic avenues for the diagnosis and management of glioma.

In this study, we conducted an analysis of gene expression patterns between glioma patients and normal samples using data from the GEO database, identifying 4092 DEGs. Among these, we pinpointed 69 differentially expressed ferroptosis-related genes in glioma. Through further scrutiny, we highlighted TP53, RRM2, EZH2, CDKN1A, MYCN, KIF20A, BLM, GLS2, HMOX1, and GOT1 as pivotal hub genes. Our enrichment analysis revealed that the dysregulation of ferroptosis pathways in glioma is intricately linked to cellular apoptosis, reactive oxygen metabolism, the p53 signaling pathway, and immune modulation.

To validate the expression patterns of these hub genes, we performed qRT-PCR on glioma cells and normal human astrocytes. Consistent with the bioinformatics analysis, the qRT-PCR results demonstrated that TP53, RRM2, EZH2, CDKN1A, MYCN, KIF20A, BLM, and HMOX1 were significantly upregulated in glioma cells, whereas GLS2 and GOT1 were markedly downregulated. These findings reinforce the reliability of our computational screening and highlight the potential functional divergence among ferroptosis-related genes in glioma pathophysiology.

Ferroptosis represents a novel form of cell demise triggered by the accumulation of iron-dependent lipid peroxides. Differing from conventional cell death pathways such as apoptosis, necrosis, and autophagy, ferroptosis possesses distinct molecular features and biological processes. It hinges on intracellular iron elevation, where iron catalyzes lipid peroxidation via the Fenton reaction, generating copious reactive oxygen species (ROS) and initiating lipid peroxidation of the cell membrane. Polyunsaturated fatty acids (PUFAs) on the cell membrane serve as prime targets of ferroptosis. Under the influence of iron and ROS, PUFAs undergo peroxidation, compromising cell membrane integrity and ultimately culminating in cell demise.^[41]^ TP53, a pivotal player in various cellular functions including cell cycle regulation, DNA repair, apoptosis, and metabolism, emerges as a central mediator in ferroptosis modulation. Studies have underscored TP53’s intimate association with ferroptosis, exerting its influence through diverse pathways. TP53 can boost SLC7A11 expression (the gene encoding xCT protein), impede glutamate-cysteine exchange, influence glutathione synthesis, thereby fine-tuning ferroptosis. Moreover, TP53 orchestrates the oxidative stress response by regulating lipid synthesis-associated genes (e.g., ACSL4 and LPCAT3), thus modulating lipid peroxidation and ferroptosis.^[42]^ Although the precise mechanism underlying ferroptosis in glioma etiology and progression remains elusive, our findings delineate distinct expression patterns of key ferroptosis-related genes in gliomas. Elevated expression of key ferroptosis genes in the risk model portends a favorable prognosis. In essence, ferroptosis may wield significant sway in tumor initiation, progression, and therapeutic interventions, with the modulation of ferroptosis-associated pathways presenting novel avenues for tumor management. TP53 emerges as a pivotal target for ferroptosis regulation in both preventive and therapeutic endeavors. Although the precise mechanisms through which ferroptosis contributes to glioma development remain to be fully elucidated, our findings suggest that ferroptosis-related genes exhibit distinct expression profiles and may play divergent roles in tumor biology. These genes may serve as promising biomarkers and therapeutic targets for glioma management. TP53, in particular, emerges as a pivotal candidate for ferroptosis modulation in both preventive and therapeutic strategies.

The tumor microenvironment (TME) constitutes a multifaceted milieu comprising tumor cells, immune cells, stromal cells, blood vessels, and various signaling molecules. Immune infiltration denotes the infiltration of immune cells into the TME, exerting a pivotal influence on tumor initiation, progression, and treatment response. Emerging evidence underscores the intimate interplay between ferroptosis and immune infiltration. Cellular debris and metabolites released during ferroptosis, such as high-mobility group protein B1, can function as antigens, thereby enticing and activating dendritic cells and macrophages, bolstering antitumor immune responses.^[43]^ Additionally, lipid peroxides and reactive oxygen species (ROS) generated during ferroptosis can incite inflammatory responses and attract immune cells like neutrophils, macrophages, and lymphocytes into the TME.^[44]^ Furthermore, ferroptosis exerts regulatory control over immunity by modulating T cell function and influencing macrophage polarization.^[45]^ Our study revealed a heightened state of immune infiltration in gliomas through immune infiltration analysis. Notably, plasma cells, CD8 T cells, follicular helper T cells, activated NK cells, and macrophages exhibited significant augmentation in glioma tissues. Plasma cells, arising from B cells differentiation, orchestrate immune responses primarily mediated by antibodies.^[46]^ CD8+ T cells, the principal cytotoxic T lymphocytes, recognize and engage antigens on tumor cell surfaces, releasing perforin and granzymes to directly obliterate tumor cells. They also secrete an array of cytokines, such as IFN-γ, to potentiate the antitumor activity of other immune cell populations including macrophages and NK cells.^[47]^ Similarly, follicular helper T cells, activated NK cells, and macrophages wield multifaceted and pivotal roles within TME.^[48-50]^ Immune evasion constitutes a pivotal hallmark driving tumor development. Our study underscores the close nexus between immune response and glioma occurrence, progression, and therapeutic response, positing immune response modulation as a promising avenue for glioma treatment intervention.

Based on the prediction of drug targets derived from hub genes, we conducted a preliminary analysis of 43 potential compounds. Tazemetostat, for instance, functions as a methyltransferase inhibitor that specifically targets EZH2. By inhibiting EZH2, Tazemetostat prevents excessive trimethylation of histone H3 at lysine 27 (H3K27), thereby impeding uncontrolled cell cycle progression and inhibiting the dedifferentiation of cancer cells.^[51]^ Imexon, on the other hand, acts as a thiol-binding small molecule that penetrates cells and binds to glutathione and other thiol compounds. By binding to these molecules, Imexon prevents them from scavenging toxic free radicals. Consequently, in rapidly dividing cancer cells, the accumulation of free radicals ensues, leading to alterations in mitochondrial membrane potential. Ultimately, this cascade of events culminates in mitochondrial swelling and rupture, with the release of mitochondrial proteins – especially cytochrome *c* – into the cytoplasm. This triggers caspase-mediated apoptosis, culminating in cancer cell demise.^[52]^ Nintedanib operates by inhibiting kinase signaling pathways in various cell types within tumor tissues, including endothelial cells, pericytes, smooth muscle cells, and cells involved in angiogenesis. By impeding these pathways, Nintedanib curtails cell proliferation and induces apoptosis in affected tumor cells.^[53]^ Similarly, Motexafin gadolinium, Ponatinib, and Regorafenib have demonstrated inhibitory effects on tumors.^[54-56]^ Collectively, the drug prediction based on hub gene targets suggests that Tazemetostat, Imexon, Motexafin gadolinium, Ponatinib, Nintedanib, and Regorafenib hold promise as key drugs or ingredients for glioma treatment. These findings may offer valuable insights for clinical management strategies aimed at combating gliomas.

Although our study provides valuable insights into the role of ferroptosis-related genes in glioma and highlights potential therapeutic targets, several limitations should be acknowledged. First, the identification of differentially expressed ferroptosis-related genes and hub targets was predominantly based on publicly available transcriptomic datasets, which, while informative, require further validation through in vivo and in vitro experiments. Second, although we performed qRT-PCR to validate gene expression, the experimental data were derived from a single glioma cell line, which may not fully capture the heterogeneity of glioma subtypes. Future studies involving multiple glioma cell lines and patient-derived samples are warranted to enhance the generalizability of our findings. Third, the therapeutic compounds predicted based on hub gene interactions remain hypothetical; their efficacy and mechanisms of action must be rigorously tested through pharmacological and functional assays before clinical translation.

## 5. Conclusion

In conclusion, our study has yielded valuable insights into glioma pathogenesis and potential therapeutic strategies. By establishing a disease prediction model, we have highlighted the intricate interplay between glioma and key signaling pathways such as ferroptosis, apoptosis, reactive oxygen metabolism, the p53 signaling pathway, and immune regulation. This enhanced understanding of glioma development mechanisms paves the way for more targeted and effective treatment approaches. Furthermore, our prediction of Tazemetostat, Imexon, Motexafin gadolinium, Ponatinib, Nintedanib, and Regorafenib as potential key drugs or ingredients for glioma treatment offers promising avenues for clinical intervention. These findings hold significant potential for improving patient outcomes and advancing the field of glioma therapy. In summary, our research provides novel insights, directions, and targets for the clinical management of glioma. We believe that these findings will contribute to the development of more effective therapeutic strategies and ultimately improve the prognosis and quality of life for glioma patients.

## Author contributions

**Formal analysis:** Dan Wu.

**Software:** Dan Wu.

**Writing – original draft:** Yang Zhang.

**Writing – review & editing:** Yingjiang Gu, Hua Xiao.

## Supplementary Material



## References

[1] Ostrom QT Price M Neff C. CBTRUS statistical report: primary brain and other central nervous system tumors diagnosed in the United States in 2015-2019. Neuro Oncol. 2022;24(Suppl 5):v1–v95. 10.1093/neuonc/noac202 PMC9533228 36196752

[2] Francis SS Ostrom QT Cote DJ Smith TR Claus E Barnholtz-Sloan JS. The epidemiology of central nervous system tumors. Hematol Oncol Clin North Am. 2022;36:23–42. 10.1016/j.hoc.2021.08.012 34801162

[3] Zeng Z Chen Y Geng X. NcRNAs: multi‑angle participation in the regulation of glioma chemotherapy resistance (Review). Int J Oncol. 2022;60:76. 10.3892/ijo.2022.5366 PMC9083885 35506469

[4] Schwartzbaum JA Fisher JL Aldape KD Wrensch M. Epidemiology and molecular pathology of glioma. Nat Clin Pract Neurol. 2006;2:494–503; quiz 1 p following 516. 10.1038/ncpneuro0289 16932614

[5] Edick MJ Cheng C Yang W. Lymphoid gene expression as a predictor of risk of secondary brain tumors. Genes Chromosomes Cancer. 2005;42:107–16. 10.1002/gcc.20121 15543619

[6] Ohgaki H Kleihues P. Epidemiology and etiology of gliomas. Acta Neuropathol. 2005;109:93–108. 10.1007/s00401-005-0991-y 15685439

[7] Samanic CM Cote DJ Creed JH. Prospective study of sleep duration and glioma risk. Cancer Causes Control. 2021;32:1039–42. 10.1007/s10552-021-01447-9 PMC8859696 34014383

[8] Yoshikawa MH Rabelo NN Telles JPM Figueiredo EG. Modifiable risk factors for glioblastoma: a systematic review and meta-analysis. Neurosurg Rev. 2023;46:143. 10.1007/s10143-023-02051-y 37340151

[9] Tan Y Mu W Wang XC Yang GQ Gillies RJ Zhang H. Improving survival prediction of high-grade glioma via machine learning techniques based on MRI radiomic, genetic and clinical risk factors. Eur J Radiol. 2019;120:108609. 10.1016/j.ejrad.2019.07.010 31606714

[10] Zhou S Wang X Tan Y Qiu L Fang H Li W. Association between vitamin C intake and glioma risk: evidence from a meta-analysis. Neuroepidemiology. 2015;44:39–44. 10.1159/000369814 25720916

[11] Raysi Dehcordi S Galzio R Marrone F. Brain location and tumor biological markers in high- and low-grade gliomas. J Neurosurg Sci. 2023;67:143–49. 10.23736/S0390-5616.20.05047-X 33320464

[12] Hou P Wan Q Wang Q Wu X Lu X. Overexpression of RAB34 associates with tumor aggressiveness and immune infiltration in glioma. Biosci Rep. 2022;42:BSR20212624. 10.1042/BSR20212624 PMC9620491 36222286

[13] Zhang N Zhang H Wu W. Machine learning-based identification of tumor-infiltrating immune cell-associated lncRNAs for improving outcomes and immunotherapy responses in patients with low-grade glioma. Theranostics. 2022;12:5931–48. 10.7150/thno.74281 PMC9373811 35966587

[14] Marinari E Allard M Gustave R. Inflammation and lymphocyte infiltration are associated with shorter survival in patients with high-grade glioma. Oncoimmunology. 2020;9:1779990. 10.1080/2162402X.2020.1779990 PMC7458651 32923142

[15] Guo Z Su Z Wei Y Zhang X Hong X. Pyroptosis in glioma: current management and future application. Immunol Rev. 2024;321:152–68. 10.1111/imr.13294 38063042

[16] Luo Y Tian G Fang X Bai S Yuan G Pan Y. Ferroptosis and its potential role in glioma: from molecular mechanisms to therapeutic opportunities. Antioxidants (Basel). 2022;11:2123. 10.3390/antiox11112123 PMC9686959 36358495

[17] Yang WS Stockwell BR. Ferroptosis: death by lipid peroxidation. Trends Cell Biol. 2016;26:165–76. 10.1016/j.tcb.2015.10.014 PMC4764384 26653790

[18] Hao S Liang B Huang Q. Metabolic networks in ferroptosis. Oncol Lett. 2018;15:5405–11. 10.3892/ol.2018.8066 PMC5844144 29556292

[19] Cao JY Dixon SJ. Mechanisms of ferroptosis. Cell Mol Life Sci. 2016;73:2195–209. 10.1007/s00018-016-2194-1 PMC4887533 27048822

[20] Tian Y Lu J Hao X. FTH1 inhibits ferroptosis through ferritinophagy in the 6-OHDA model of parkinson’s disease. Neurotherapeutics. 2020;17:1796–812. 10.1007/s13311-020-00929-z PMC7851296 32959272

[21] Lu B Chen XB Ying MD He QJ Cao J Yang B. The role of ferroptosis in cancer development and treatment response. Front Pharmacol. 2017;8:992. 10.3389/fphar.2017.00992 PMC5770584 29375387

[22] Yuan S Xi S Weng H. YTHDC1 as a tumor progression suppressor through modulating FSP1-dependent ferroptosis suppression in lung cancer. Cell Death Differ. 2023;30:2477–90. 10.1038/s41418-023-01234-w PMC10733405 37903990

[23] Hu R Shi Z Yang J Ren Y Li X. Anti-ferroptosis: a promising therapeutic method for thyroid cancer. Front Biosci (Landmark Ed). 2024;29:77. 10.31083/j.fbl2902077 38420811

[24] Liu T Zhu C Chen X. Ferroptosis, as the most enriched programmed cell death process in glioma, induces immunosuppression and immunotherapy resistance. Neuro Oncol. 2022;24:1113–25. 10.1093/neuonc/noac033 PMC9248406 35148413

[25] Chen S Zhang Z Zhang B. CircCDK14 promotes tumor progression and resists ferroptosis in glioma by regulating PDGFRA. Int J Biol Sci. 2022;18:841–57. 10.7150/ijbs.66114 PMC8741855 35002529

[26] Sim AY Minary P Levitt M. Modeling nucleic acids. Curr Opin Struct Biol. 2012;22:273–8. 10.1016/j.sbi.2012.03.012 PMC4028509 22538125

[27] Dawson WK Maciejczyk M Jankowska EJ Bujnicki JM. Coarse-grained modeling of RNA 3D structure. Methods. 2016;103:138–56. 10.1016/j.ymeth.2016.04.026 27125734

[28] Kmiecik S Gront D Kolinski M Wieteska L Dawid AE Kolinski A. Coarse-grained protein models and their applications. Chem Rev. 2016;116:7898–936. 10.1021/acs.chemrev.6b00163 27333362

[29] Taminau J Meganck S Lazar C. Unlocking the potential of publicly available microarray data using inSilicoDb and inSilicoMerging R/Bioconductor packages. BMC Bioinf. 2012;13:335. 10.1186/1471-2105-13-335 PMC3568420 23259851

[30] Johnson WE Li C Rabinovic A. Adjusting batch effects in microarray expression data using empirical Bayes methods. Biostatistics. 2007;8:118–27. 10.1093/biostatistics/kxj037 16632515

[31] Ritchie ME Phipson B Wu D. limma powers differential expression analyses for RNA-sequencing and microarray studies. Nucleic Acids Res. 2015;43:e47. 10.1093/nar/gkv007 PMC4402510 25605792

[32] Yuan WH Xie QQ Wang KP. Screening of osteoarthritis diagnostic markers based on immune-related genes and immune infiltration. Sci Rep. 2021;11:7032. 10.1038/s41598-021-86319-7 PMC8007625 33782454

[33] Ye L Liang R Liu X Li J Yue J Zhang X. Frailty and sarcopenia: a bibliometric analysis of their association and potential targets for intervention. Ageing Res Rev. 2023;92:102111. 10.1016/j.arr.2023.102111 38031836

[34] van de Velden M D’Enza AI Palumbo F. Cluster correspondence analysis. Psychometrika. 2017;82:158–85. 10.1007/s11336-016-9514-0 27683298

[35] Shannon P Markiel A Ozier O. Cytoscape: a software environment for integrated models of biomolecular interaction networks. Genome Res. 2003;13:2498–504. 10.1101/gr.1239303 PMC403769 14597658

[36] Otasek D Morris JH Bouças J Pico AR Demchak B. Cytoscape automation: empowering workflow-based network analysis. Genome Biol. 2019;20:185. 10.1186/s13059-019-1758-4 PMC6717989 31477170

[37] Babbi G Martelli PL Profiti G Bovo S Savojardo C Casadio R. eDGAR: a database of disease-gene associations with annotated relationships among genes. BMC Genomics. 2017;18(Suppl 5):554. 10.1186/s12864-017-3911-3 PMC5558190 28812536

[38] Eraso-Pichot A Brasó-Vives M Golbano A. GSEA of mouse and human mitochondriomes reveals fatty acid oxidation in astrocytes. Glia. 2018;66:1724–35. 10.1002/glia.23330 29575211

[39] Kim Y Kang JW Kang J. Novel deep learning-based survival prediction for oral cancer by analyzing tumor-infiltrating lymphocyte profiles through CIBERSORT. Oncoimmunology. 2021;10:1904573. 10.1080/2162402X.2021.1904573 PMC8018482 33854823

[40] Le Rhun E Preusser M Roth P. Molecular targeted therapy of glioblastoma. Cancer Treat Rev. 2019;80:101896. 10.1016/j.ctrv.2019.101896 31541850

[41] Jiang X Stockwell BR Conrad M. Ferroptosis: mechanisms, biology and role in disease. Nat Rev Mol Cell Biol. 2021;22:266–82. 10.1038/s41580-020-00324-8 PMC8142022 33495651

[42] Kang R Kroemer G Tang D. The tumor suppressor protein p53 and the ferroptosis network. Free Radic Biol Med. 2019;133:162–8. 10.1016/j.freeradbiomed.2018.05.074 PMC6251771 29800655

[43] Tang R Xu J Zhang B. Ferroptosis, necroptosis, and pyroptosis in anticancer immunity. J Hematol Oncol. 2020;13:110. 10.1186/s13045-020-00946-7 PMC7418434 32778143

[44] Chen X Kang R Kroemer G Tang D. Ferroptosis in infection, inflammation, and immunity. J Exp Med. 2021;218:e20210518. 10.1084/jem.20210518 PMC8126980 33978684

[45] Gong C Ji Q Wu M. Ferroptosis in tumor immunity and therapy. J Cell Mol Med. 2022;26:5565–79. 10.1111/jcmm.17529 PMC9667519 36317423

[46] Cancro MP Tomayko MM. Memory B cells and plasma cells: the differentiative continuum of humoral immunity. Immunol Rev. 2021;303:72–82. 10.1111/imr.13016 34396546

[47] Baharom F Ramirez-Valdez RA Khalilnezhad A. Systemic vaccination induces CD8(+) T cells and remodels the tumor microenvironment. Cell. 2022;185:4317–32.e15. 10.1016/j.cell.2022.10.006 PMC9669246 36302380

[48] Overacre-Delgoffe AE Bumgarner HJ Cillo AR. Microbiota-specific T follicular helper cells drive tertiary lymphoid structures and anti-tumor immunity against colorectal cancer. Immunity. 2021;54:2812–24.e4. 10.1016/j.immuni.2021.11.003 PMC8865366 34861182

[49] Wu SY Fu T Jiang YZ Shao ZM. Natural killer cells in cancer biology and therapy. Mol Cancer. 2020;19:120. 10.1186/s12943-020-01238-x PMC7409673 32762681

[50] Sheu KM Hoffmann A. Functional hallmarks of healthy macrophage responses: their regulatory basis and disease relevance. Annu Rev Immunol. 2022;40:295–321. 10.1146/annurev-immunol-101320-031555 PMC10074967 35471841

[51] Italiano A Soria JC Toulmonde M. Tazemetostat, an EZH2 inhibitor, in relapsed or refractory B-cell non-Hodgkin lymphoma and advanced solid tumours: a first-in-human, open-label, phase 1 study. Lancet Oncol. 2018;19:649–59. 10.1016/S1470-2045(18)30145-1 29650362

[52] Evens AM Prachand S Shi B Paniaqua M Gordon LI Gartenhaus RB. Imexon-induced apoptosis in multiple myeloma tumor cells is caspase-8 dependent. Clin Cancer Res. 2004;10:1481–91. 10.1158/1078-0432.ccr-1058-03 14977852

[53] Tu J Xu H Ma L. Nintedanib enhances the efficacy of PD-L1 blockade by upregulating MHC-I and PD-L1 expression in tumor cells. Theranostics. 2022;12:747–66. 10.7150/thno.65828 PMC8692903 34976211

[54] Magda D Miller RA. Motexafin gadolinium: a novel redox active drug for cancer therapy. Semin Cancer Biol. 2006;16:466–76. 10.1016/j.semcancer.2006.09.002 17112739

[55] Gao Y Ding Y Tai XR Zhang C Wang D. Ponatinib: an update on its drug targets, therapeutic potential and safety. Biochim Biophys Acta Rev Cancer. 2023;1878:188949. 10.1016/j.bbcan.2023.188949 37399979

[56] Strumberg D Schultheis B. Regorafenib for cancer. Expert Opin Investig Drugs. 2012;21:879–89. 10.1517/13543784.2012.684752 22577890

